# Supplementary material for: A dual role of EZH2 in regulating A-to-I RNA editing and mRNA stability through ADAR
Source: Nat Commun. 2026 Mar 26;17:4421. doi: 10.1038/s41467-026-71207-3 (PMC13184275; doi:10.1038/s41467-026-71207-3)
Supplement: Supplementary file 1 — Supplementary Information [file 41467_2026_71207_MOESM1_ESM.pdf]

Supplementary materials

A dual role of EZH2 in regulating A-to-I RNA editing and mRNA stability through ADAR

Yang Yi, Yanqiang Li, Rui Wang, Xufen Yu, Qi Liu, Chaehyun Yum, Yang Zhang, Yuanyuan Qiao, Aileen Szczepanski, Siqi Wu, Qiaqia Li, Ladan Fazli, Jiangchuan Shen, Xin Wang, Xiaoling Li, Ping Mu, Edward M. Schaeffer, Heather A. Hundley, Hengyao Niu, Arul M. Chinnaiyan, Lu Wang, Jinjun Shi, Jian Jin, Xuesen Dong, Wei Zhao, Kaifu Chen, Qi Cao

Contents

Supplementary Figures 1-8

Supplementary Table 1-3

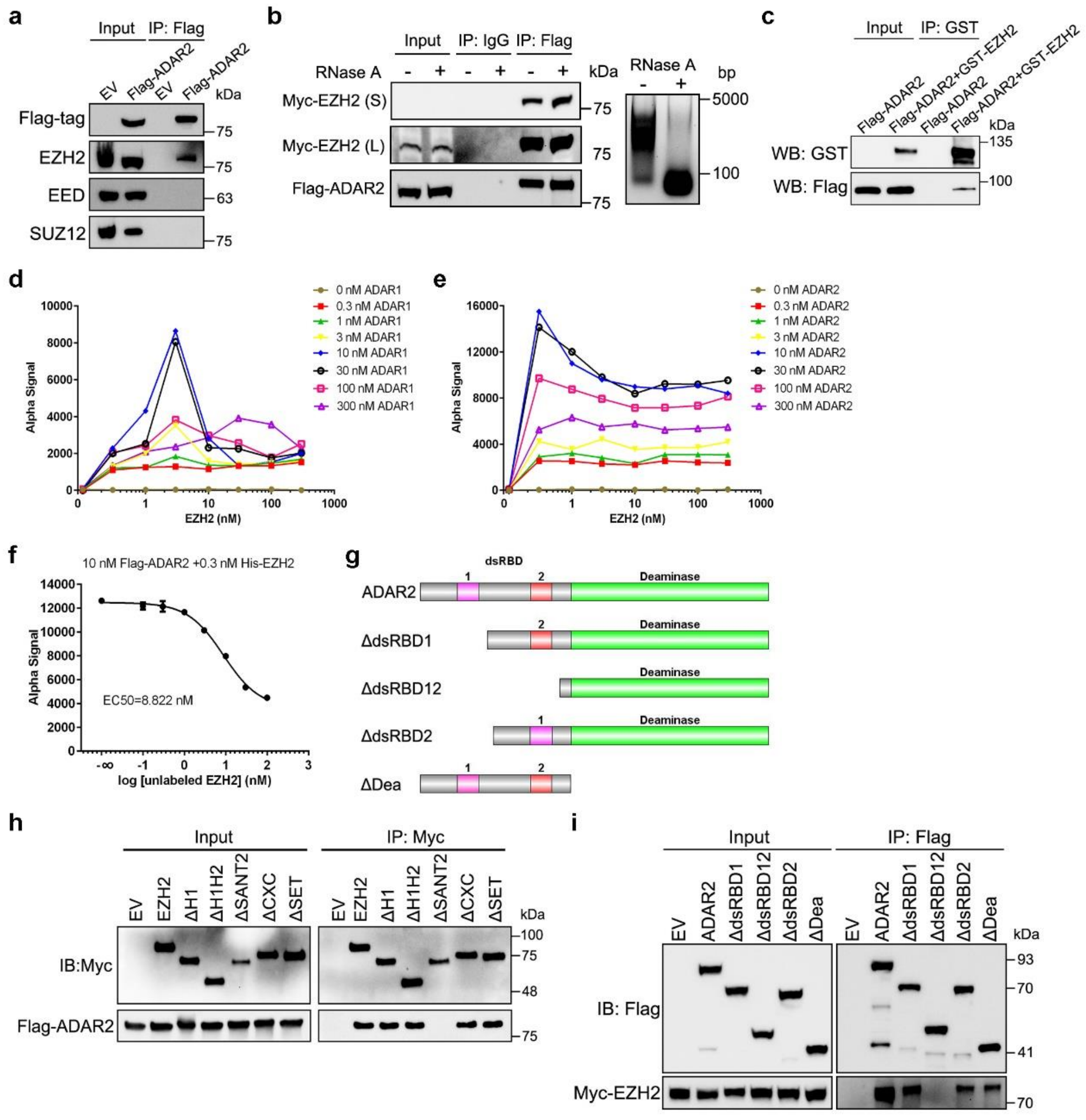

Supplementary Figure 1. Both ADAR1 and ADAR2 directly bind to EZH2.

(a) C4-2 cells overexpressing empty vector (EV) or Flag-tagged ADAR2 were lysed and subjected to co-IP assay using anti-Flag antibody, followed by western blot with indicated antibodies.

(b) C4-2 cells co-transfected with Myc-tagged EZH2 and Flag-tagged ADAR2 were lysed and subjected to co-IP assay using anti-Flag antibody. Before IP, cell lysates were treated with or without RNase A. Both the shorter (S) and longer (L) exposures

of EZH2 bands were presented. Agarose gel demonstrated the complete digestion of total RNA.

(c) Purified proteins of GST-tagged EZH2 and Flag-tagged ADAR2 were subjected to GST pull down assay, followed by western blot with indicated antibodies.

(d) AlphaLISA cross-titration assay to determine the optimal protein concentration combination of EZH2 and ADAR1p110. A hook point is reached at 3 nM His-tagged EZH2 and 10 nM Flag-tagged ADAR1p110.

(e) AlphaLISA cross-titration assay to determine the optimal protein concentration combination of EZH2 and ADAR2. A hook point is reached at 0.3 nM His-tagged EZH2 and 10 nM Flag-tagged ADAR2.

(f) Inhibition of His-tagged EZH2 and Flag-tagged ADAR2 binding by unlabeled EZH2 in AlphaLISA displacement assay. Data represent Mean ± SD for n=3 biologically independent experiments.

(g) Schematic diagrams of ADAR2 protein and its truncation mutants.

(h) Co-IP of Flag-tagged ADAR2 with full-length or truncation mutants of Myc-tagged EZH2, followed by western blot with indicated antibodies.

(i) Co-IP of Myc-tagged EZH2 with full-length or truncation mutants of Flag-tagged ADAR2, followed by western blot with indicated antibodies.

Source data are provided as a Source Data file.

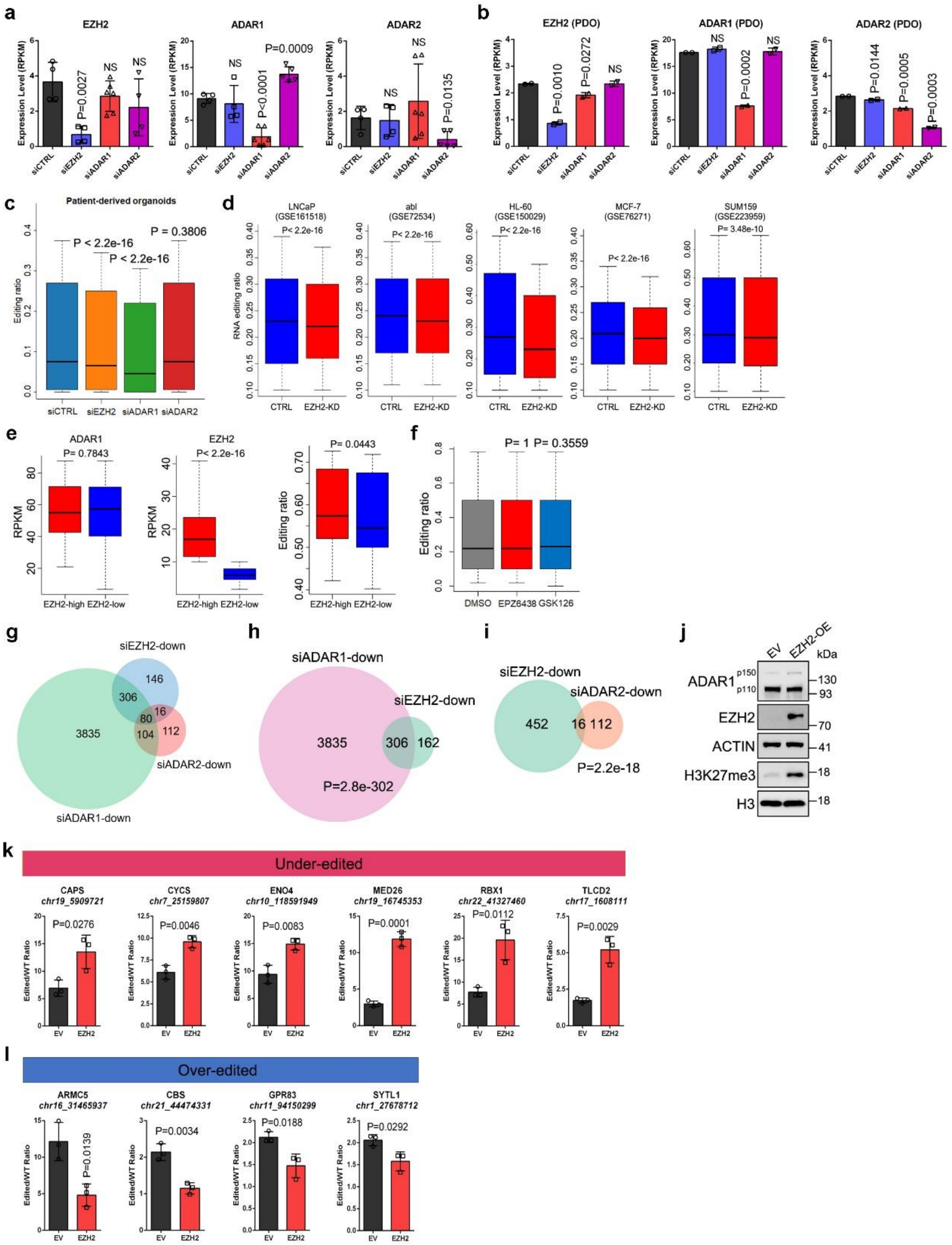

Supplementary Figure 2. EZH2 modulates A-to-I editing pattern globally in PCa cells.

(a) Graph showing the expression levels of EZH2, ADAR1 and ADAR2 in each group, as profiled by RNA-seq in C4-2 cells. RPKM, reads per kilobase of transcript per million reads mapped.

(b) Graph showing the expression levels of EZH2, ADAR1 and ADAR2 in each group, as profiled by RNA-seq using human PCa PDO MDA-PCa-174.

(c) Box plot showing the global editing ratio in each PDO group. P values were calculated by two-tailed unpaired Wilcoxon's test.

(d) Box plots showing the change of editing ratio upon EZH2 knockdown (KD) in cancer cell lines. LNCaP and abl are PCa cell lines, HL-60 is Leukemia cell line, while MCF-7 and SUM159 are breast cancer cell lines. P values were calculated by two-tailed unpaired Wilcoxon's test.

(e) PCa samples from SU2C were divided into EZH2-high and -low groups (n=88 for each). The RPKM values of ADAR1 and EZH2 along with the editing ratio were presented. P values were calculated by two-tailed unpaired Wilcoxon's test.

(f) Box plot showing the global editing ratio in each group of C4-2 cells (n=4 for each). A total of 38,939 editing sites were included. P values were calculated by two-tailed unpaired Wilcoxon's test.

(g) Venn diagram showing the overlap between the under-edited sites upon ADAR1, ADAR2, and EZH2 depletion in C4-2 cells.

(h) Venn diagram showing the overlap between the independent under-edited sites upon ADAR1 and EZH2 depletion in C4-2 cells. P values were calculated by one-tailed Fisher's exact test.

(i) Venn diagram showing the overlap between the independent under-edited sites upon ADAR2 and EZH2 depletion in C4-2 cells. P values were calculated by one-tailed Fisher's exact test.

(j) Western blot to detect EZH2 protein level in PrEC upon EZH2 overexpression (OE).

(k, l) Representative RESSq-PCR results to validate the relative editing changes of EZH2-affected under-edited (**k**) and over-edited (**l**) sites in PrEC upon EZH2 overexpression. Data represent Mean ± SD from n=3 biologically independent experiments.

Statistical significance was assessed using two-sided student's t-test unless otherwise stated. Source data are provided as a Source Data file.

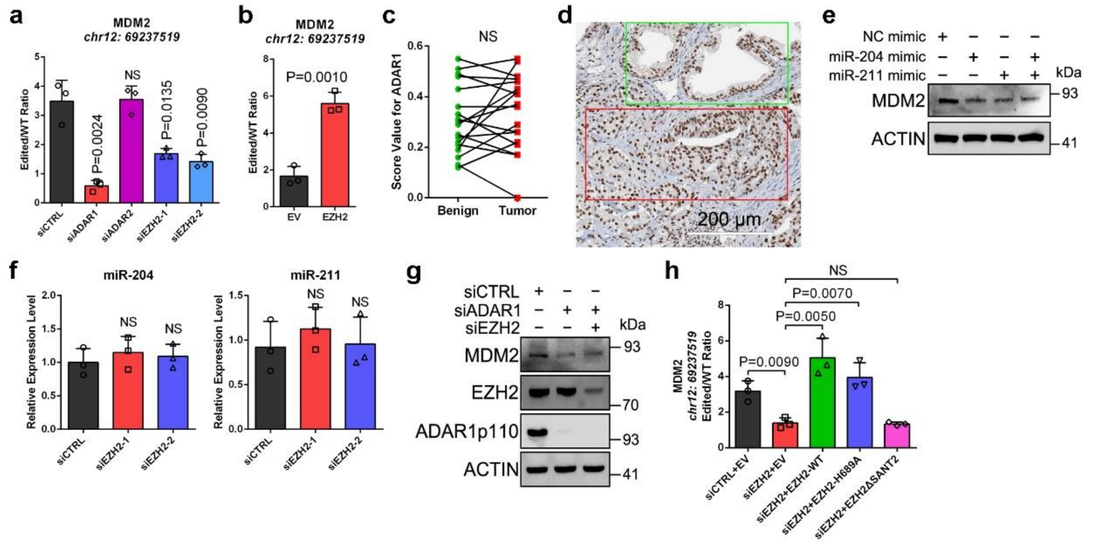

Supplementary Figure 3. EZH2-mediated RNA editing of *chr12: 69237519* regulates MDM2 expression.

(a, b) RESSq-PCR assay to monitor the relative changes of *chr12: 69237519* editing level in C4-2 cells undergoing ADAR1 or EZH2 suppression (**a**), and in PrEC with EZH2 overexpression (**b**).

(c) Paired dot plot to show the ADAR1 protein scores based on the IHC results from 18 PCa tumor samples along with the adjacent benign tissues.

(d) Representative IHC image of a PCa slide with ADAR1 staining. The red box indicates the tumor area while the green box indicates the scope of benign tissue. Scale bar: 200 µm.

(e) Western blot to detect the protein level of MDM2 in C4-2 cells upon forced expression of miR-204/miR-211 mimics or combined. NC, negative control.

(f) TaqMan qPCR analysis to detect the change of miR-204 or miR-211 expression upon EZH2 knockdown in C4-2 cells.

(g) Western blot in C4-2 cells to detect the protein level of MDM2 in each indicated group.

(h) RESSq-PCR assay to show the rescue effects of a series of EZH2 mutants on relative editing ratio of *chr12: 69237519* affected by EZH2 depletion. Graph showing the quantification of editing level in each group. Data represent Mean ± SD from n=3 biologically independent experiments.

Statistical significance was assessed using two-sided student's t-test unless otherwise stated. Source data are provided as a Source Data file.

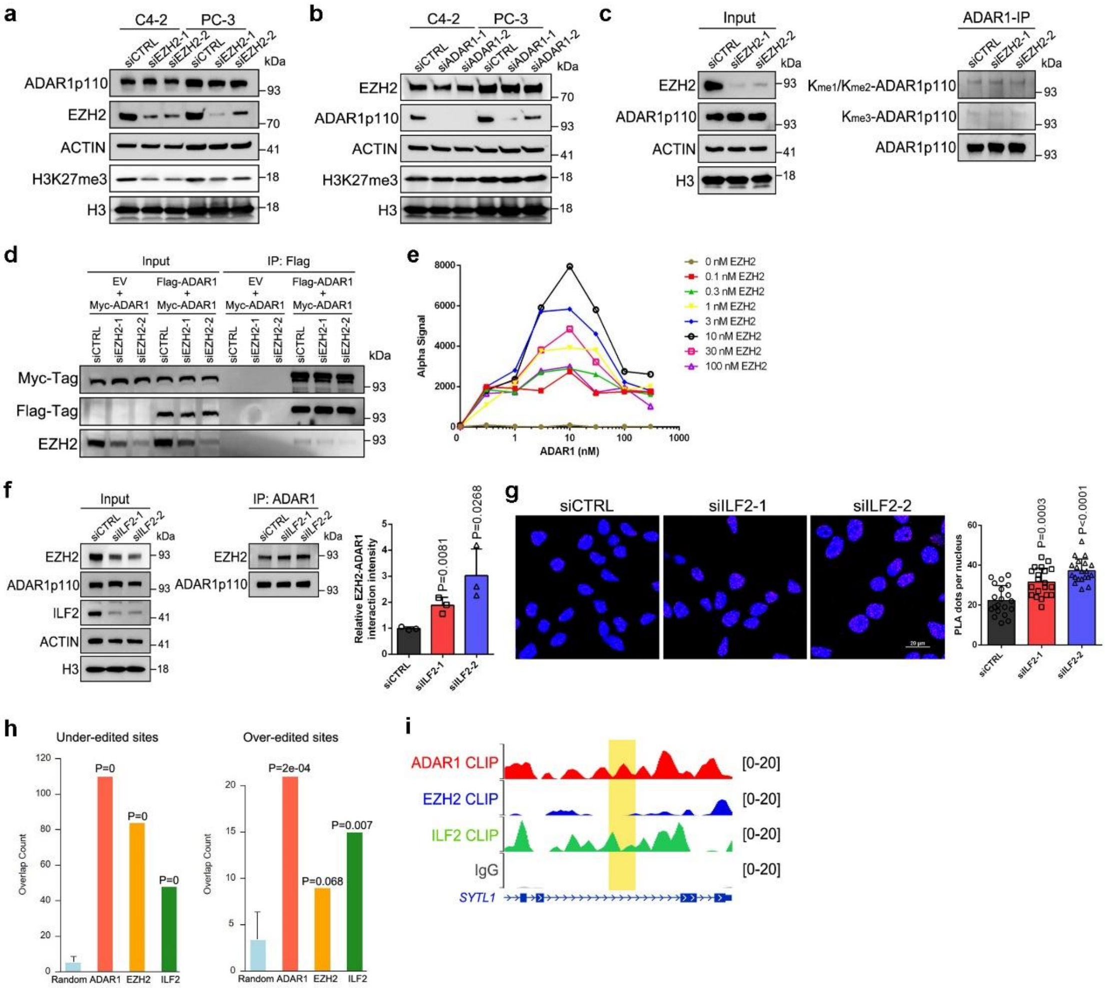

Supplementary Figure 4. EZH2 affects the editing site selectivity, but not the other characteristics of ADAR1.

(a) Western blot to detect ADAR1p110 protein level in PCa cell lines upon EZH2 knockdown.

(b) Western blot to detect EZH2 and H3K27me3 levels in PCa cell lines upon ADAR1 knockdown.

(c) Equal amount of ADAR1p110 protein was pulled down from control and EZH2-deficient C4-2 cells, followed by western blot to detect the mono-/di-methyllysine (K_{me1}/K_{me2}) and tri-methyllysine (K_{me3}) levels.

(d) Control or EZH2-deficient C4-2 cells overexpressing both Myc-tagged ADAR1p110 and Flag-tagged ADAR1p110 or empty vector (EV) control were lysed and subjected to co-IP assay using anti-Flag antibody, followed by western blot.

(e) AlphaLISA cross-titration assay to determine the optimal protein concentration combination of EZH2 and ADAR1. A hook point is reached at 10 nM GST-tagged EZH2 and 10 nM Flag-tagged ADAR1p110.

(f) Left: co-IP of the EZH2 with ADAR1p110 in control and ILF2-deficient C4-2 cells followed by WB analysis with the indicated antibodies. Right: the graph represents the relative EZH2-ADAR1 interaction intensity in each group. Data represent the mean ± SD from n = 3 biologically independent measurements. Statistical significance was determined by two-tailed Student's t-test.

(g) Left: representative images of PLAs showing interactions between EZH2 and ADAR1 in control and ILF2-deficient C4-2 cells. Right: graph showing the number of PLA dots per nucleus by counting 20 cells from each group. Statistical significance was determined by two-tailed Student's t-test. Scale bar: 20 μm.

(h) Bar plots showing the observed overlap counts between the under-edited sites (left) / over-edited (right) sites upon EZH2 knockdown and the binding regions of ADAR1, EZH2, and ILF2 (±200 nt) determined by eCLIP-seq. For each panel, the "Random" bar represents the null distribution generated from random genomic regions matched in number and size (permutation-based sampling), shown as mean ± SD. The colored bars represent the observed overlap counts for each RBP. P-values were calculated by Permutation test.

(i) Representative genome browser tracks to show eCLIP-seq data at the loci of *SYTL1*. The region covering the editing site of *chr1_27678712* was marked. Source data are provided as a Source Data file.

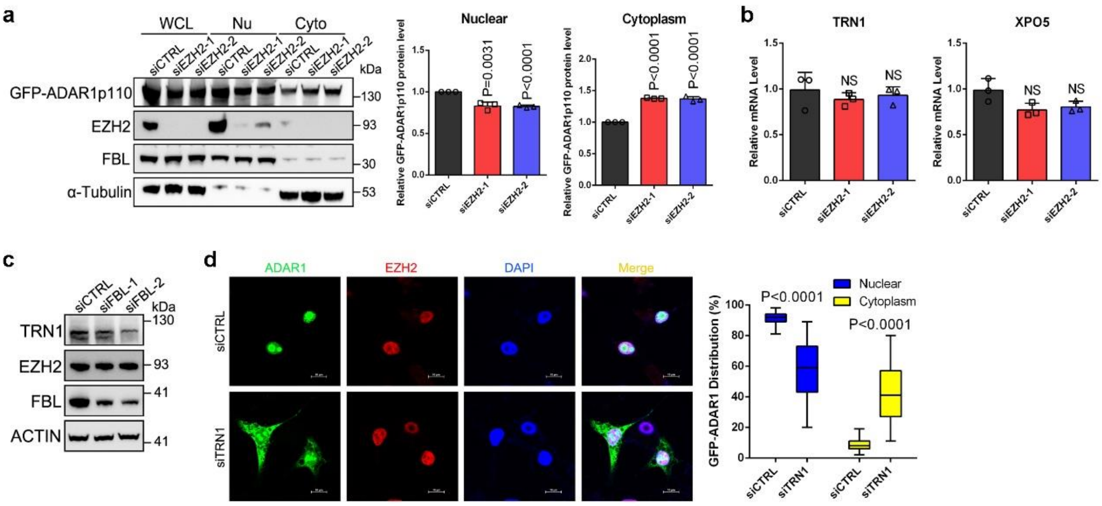

Supplementary Figure 5. EZH2 depletion induces the translocation of ADAR1p110 in a TRN1-dependent manner.

(a) Western blot to detect the distribution of exogenous ADAR1p110 proteins in whole cell lysates (WCL), nuclear (Nu) and cytoplasmic (Cyto) fractions upon EZH2 knockdown in C4-2 cells overexpressing GFP-tagged ADAR1. The graphs represent the relative ADAR1p110 protein level in each group. Data represent the mean ± s.d. from n = 3 biologically independent measurements.

(b) RT-qPCR analysis to detect the mRNA levels of TRN1 and XPO5 upon EZH2 knockdown in C4-2 cells.

(c) Western blot to detect the protein level of TRN1 upon FBL knockdown in C4-2 cells.

(d) Representative fluorescence images of control or TRN1-deficient C4-2 cells expressing GFP-tagged ADAR1p110. Endogenous EZH2 were co-stained using an anti-EZH2 antibody and the nuclei were visualized by DAPI (Scale bar: 10 μm). Graph showing the nuclear and cytoplasmic proportions of ADAR1 as estimated by the GFP intensities (Mean ± SD, n≈30).

Statistical significance was assessed using two-sided student's t-test unless otherwise stated. Source data are provided as a Source Data file.

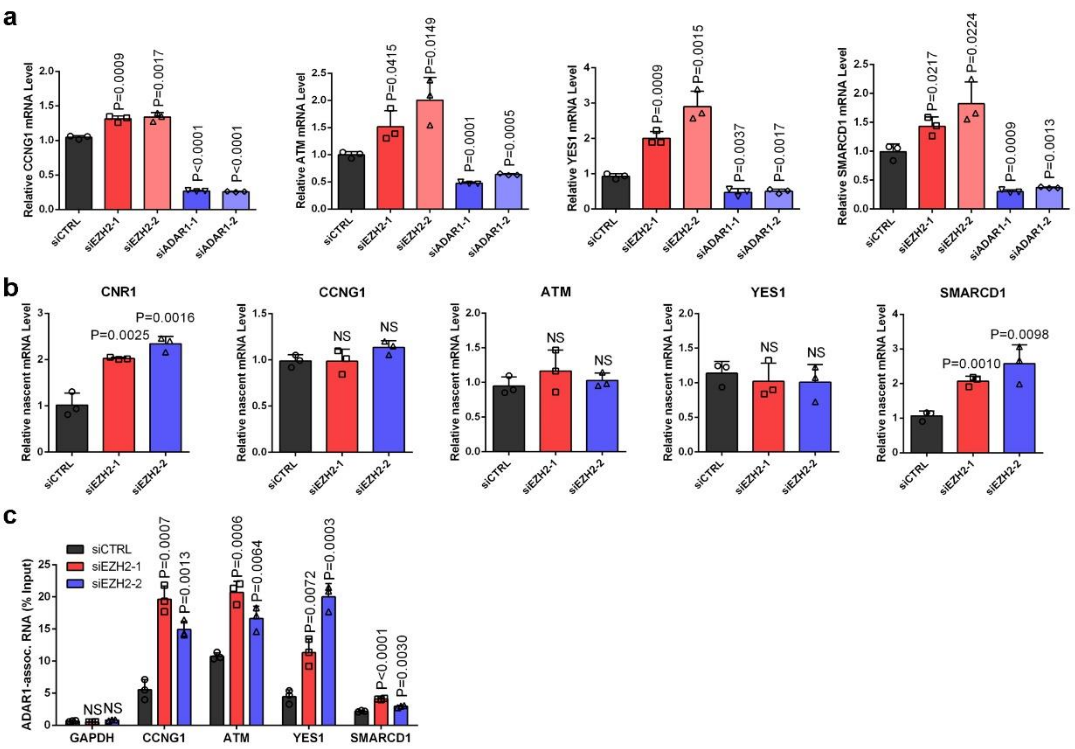

Supplementary Figure 6. Enhanced mRNA stability of selected oncogenes upon EZH2 suppression.

(a) RT-qPCR analysis to detect the ultimate mRNA level of the selected oncogenes upon EZH2 or ADAR1 knockdown in C4-2 cells.

(b) RT-qPCR analysis to detect the nascent mRNA level of the selected oncogenes upon EZH2 knockdown in C4-2 cells. CNR1, a known epigenetic target of EZH2, was used as positive control.

(c) RIP-qPCR assay to monitor the binding of selected oncogenic transcripts to ADAR1p110 upon EZH2 knockdown in C4-2 cells. GAPDH mRNA was served as negative control. Data represent the mean ± SD from n = 3 biologically independent experiments.

Statistical significance was assessed using two-sided student's t-test unless otherwise stated. Source data are provided as a Source Data file.

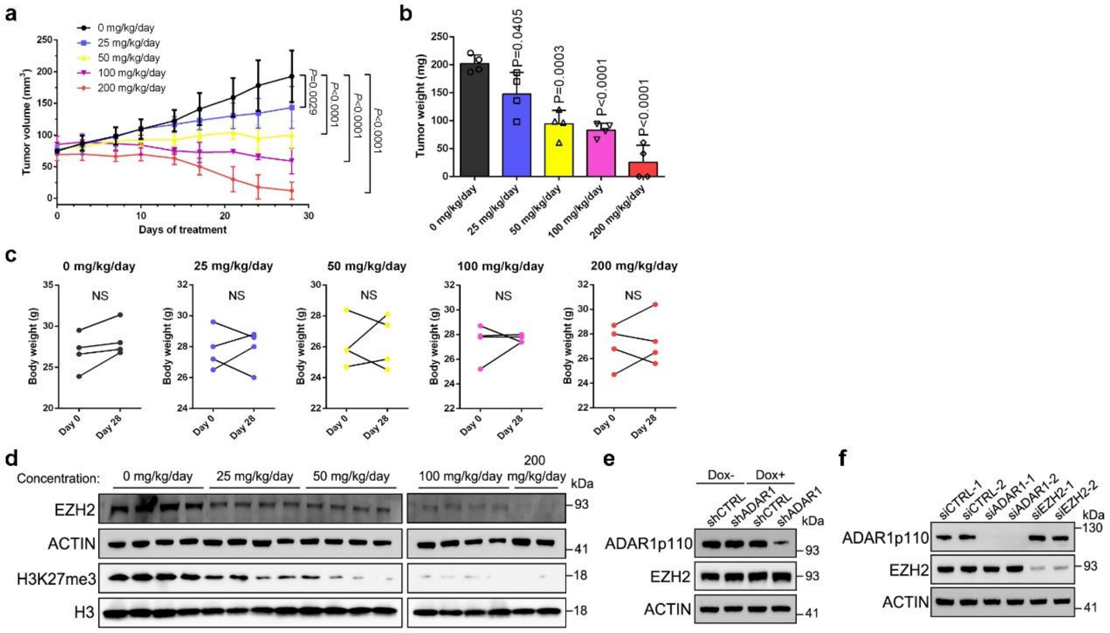

Supplementary Figure 7. EZH2 selective degrader MS8815 represses PCa growth *in vivo*.

(a-c) C4-2 cells were injected subcutaneously into SCID mice, followed by MS8815 treatment with different concentrations as indicated. Tumor volume was measured by caliper twice a week and plotted (**a**). At the end point of measurement, tumors were harvested and weighted (**b**). The body weight changes of mice during the animal experiment were summarized in **c**. Data represent Mean ± SD from n=4 mice in each group.

(d) The xenografted tumors collected above were lysed and subjected to western blot to measure EZH2 and H3K27me3 levels. For the 200 mg/kg/day group, only two tumors were grown.

(e) Western blot to verify the knockdown efficiency of ADAR1 in stable C4-2 cell lines with Dox-inducible expression of either control or ADAR1 shRNA.

(f) Western blot to verify the efficiency of ADAR1/EZH2 siRNA-containing NPs in LuCaP 35CR PDX tumors.

Statistical significance was assessed using two-sided student's t-test unless otherwise stated. Source data are provided as a Source Data file.

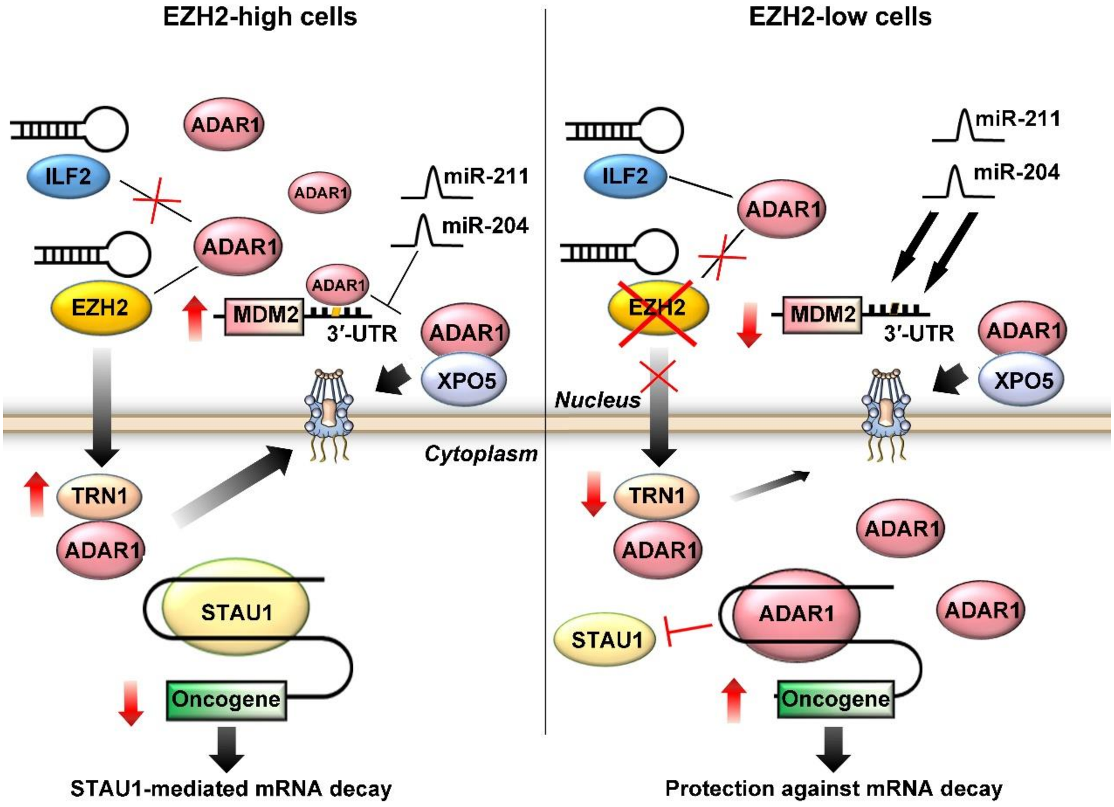

Supplementary Figure 8. A proposed model depicting the role of EZH2 in regulation of ADAR1 in PCa.

EZH2 modulates both the editing-dependent and -independent functions of ADAR1. On one hand, EZH2 interacts with ADAR1p110 directly to facilitate the editing of EZH2-bound dsRNA substrates (such as *MDM2* transcript) while inhibit the ILF2-bound RNA targets from being edited. On the other hand, EZH2 depletion downregulates TRN1 translation and subsequently leads to the accumulation of cytoplasmic ADAR1p110 to protect a number of oncogenic transcripts from STAU1-mediated RNA decay. Therefore, combinational targeting of EZH2 and ADAR1 may achieve a better therapeutic efficacy than targeting EZH2 alone for PCa treatment.

Supplementary Table 1. Antibodies used in this study.

**Antibody Target**	**Source**	**Application**
EZH2	Cell signaling, Cat#5246	WB, co-IP, IF, IHC, eCLIP
	BD, Cat#612666	PLA
ADAR1	Cell signaling, Cat #81284	WB, co-IP, PLA, IHC, eCLIP, RIP
EED	Millipore, Cat#09-774	WB (**Fig. 1a and 1b**)
	Cell signaling, Cat# 85322	WB (**Fig. 7a and 7b**)
SUZ12	Cell signaling, Cat#3737	WB
MDM2	Cell signaling, Cat#86934	WB
Histone H3	Cell signaling, Cat#4499	WB
Actin	Cell signaling, Cat#3700	WB
H3K27me3	Cell signaling, Cat#9733	WB
ILF2	Abcam, Cat#ab154791	WB, eCLIP
ILF3	Abcam, Cat#ab92355	WB
Mono/Di-methyllysine	PTM biolabs, Cat# PTM-602	WB
Tri-methyllysine	PTM biolabs, Cat# PTM-601	WB
FBL	Abcam, Cat#ab5821	WB
α-Tubulin	Cell signaling, Cat#2125	WB
TRN1	Abcam, Cat#ab10303	WB, IHC
XPO5	Abcam, Cat#ab131281	WB

CCNG1	Santa Cruz, Cat#sc-8016	WB
ATM	Cell signaling, Cat#2873	WB
YES1	Abcam, Cat#ab109265	WB
SMARCD1	Abcam, Cat#ab245222	WB
STAU1	Abcam, Cat#ab73478	RIP
Flag-tag	Cell signaling, Cat#14793	WB, co-IP
Myc-tag	Cell signaling, Cat#2278	WB, co-IP
GST-tag	Abcam, Cat#ab111947	WB
GFP-tag	Abcam, Cat#ab1218	IF

Supplementary Table 2. Oligonucleotides used for this study.

Name	Sequence (5´to 3´)	Application
GAPDH	F: GGAGCGAGATCCCTCCAAAAT	RT-qPCR, RIP-qPCR
	R: GGCTGTTGTCATACTTCTCATGG	
TRN1	F: CTGTGAGGGAGCATTTGGTG	RT-qPCR
	R: TGTGCAACATTAGAGCTTGAGTC	
XPO5	F: ATCCTGGAACACGTTGTCAAG	RT-qPCR
	R: CACTACAATTCGAGACAGAGCAT	
CCNG1	F: GAGTCTGCACACGATAATGGC	RT-qPCR, RIP-qPCR
	R: GTGCTTGGGCTGTACCTTCA	
ATM	F: ATCTGCTGCCGTCAACTAGAA	RT-qPCR, RIP-qPCR
	R: GATCTCGAATCAGGCGCTTAAA	
YES1	F: GCCTGTCAGTACAAGTGTGAG	RT-qPCR, RIP-qPCR
	R: AAAGGCGTTACCCCTGAGGAT	
SMARCD1	F: GAGACCAGGTATGTTGCCAGG	RT-qPCR, RIP-qPCR
	R: CCAGGTCGGACTGAAGGGT	
CNR1	F: TTACAACAAGTCTCTCTCGTCCT	RT-qPCR
	R: GGCTGCCGATGAAGTGGTA	
ADAR1 shRNA oligos	TOP: CCGGGCCCACTGTTATCTTCACTTTCTCGAGAAAGTGAAGATAACAGTGGGCTTTTTG	Tet-On System
	Bottom:	

	AATTCAAAAAGCCCACTGTTATCTTCACTTTCTC<br>GAGAAAGTGAAGATAACAGTGGGC	
MDM2 (*chr12: 69237519*)	Outer-F: ATCTTGATTTAGTGTTTTTCTCCCAT<br>Outer-R: AAAAAAAGAGATTCTGCTTGGTTG<br>WT-F: TCTCTGTCTGTCTCAATAAATGGCCGAA<br>Edit-R: GACCTCCACAGGTAAACTACTAATCGCC	RESS-qPCR, Sanger sequencing
UBE2CP5 (*chr19_58379153*)	Outer-F: GTGGCTCACACCTGTAACCCC<br>Outer-R: GGCACTGGCAGAGAGACAAGAA<br>WT-F: CAGCTACTCGGGAGGCTGAGGTAA<br>Edit-R: GCCTCCTGAGTTCAAGCCATCCACC	RESS-qPCR
MED26 (*chr19_16745353*)	Outer-F: GACTCCAGGCATCCACCACC<br>Outer-R: GTTACCACACAGCCCAGCAA<br>WT-F: AGCTCAAGTGACCCGCCTACCACA<br>Edit-R: TGTAATCCCAGCACTTTGAAAGACC	RESS-qPCR
TLCD2 (*chr17_1608111*)	Outer-F: ATGGTGGGTCACGCCTGTAA<br>Outer-R: CTCTTTTCATTTCTGGGTTCCTG<br>WT-F: CCGGCTACTGAGGAGGCTGAGTCA<br>Edit-R: CTCCCAGGTTCAAACGATTCTGCC	RESS-qPCR
MBD3 (*chr19_1590505*)	Outer-F: TTTGTGTCAATAGATGGGCATAACC<br>Outer-R: CGAGACCAGTCTAGTCTAGGCAACA<br>WT-F: ACCCTCTCAGCTCAGCCTCCCAACTA	RESS-qPCR

	Edit-R: TGGCACGTGCCTGTAATTCCCTCC	
TULP3 (*chr12_2996465*)	Outer-F: TGGCTGGGTGCCGTGA	RESS-qPCR
	Outer-R: GCAACCTCCGACTCCCTGAT	
	WT-F: TCAAGGTCAGCCTGGTCAACATGCTA	
	Edit-R: TTTGTATTTTTAGTAGAGACAGGATGTC	
ZNF587 (*chr19_58355170*)	Outer-F: TTTCACTGTCACCGATACTGGAG	RESS-qPCR
	Outer-R: CACAGCAAGACCCTGTCTCTACA	
	WT-F: GTACTTTTAGTAGAGATGGGGTTTCAGTA	
	Edit-R: GGAGTTCAAGATCAGTTTGACCAAGAC	
RBX1 (*chr22_41327460*)	Outer-F: GGTTAGATCATTAACAATACCACCTAAA	RESS-qPCR
	Outer-R: TCCTCCCACCTCAGCCTCC	
	WT-F: AAGTTGGGTGCAGTGGATCACAGGTA	
	Edit-R: GTCTCCCAAAGTGCTGGGATGAC	
ZNF708 (*chr19_21474482*)	Outer-F: GCCTCCTGGGTTCAAGCAA	RESS-qPCR
	Outer-R: GCATCAAGTAACTAGAGAGTTGAATCAC	
	WT-F: TTTAGTAGGGACGGGGTTTCACCGTA	
	Edit-R: AGGAGTTCAAGACTACCCTGACCGAC	
GSEC (*chr11_126216862*)	Outer-F: TCATTCAGAGATTAGCCCCTTTAC	RESS-qPCR
	Outer-R: CCCTGAGGCTTTCTGACCAC	
	WT-F: CAGGTGTGGTGGTGGGCTCGTA	
	Edit-R: TCAGCCTCCCAAGTAGGTGGGATAAC	
TFDP2	Outer-F: CAGTGCCTCTCGCCTGTAATC	RESS-qPCR

(*chr3_141668831*)	Outer-R: TCTACAACCTCCGCCTCCTG	
	WT-F: GGCAACATGACAAAACCCTGTCTGTA	
	Edit-R: CACACCCAGCTAATTTTTGTATTTTCGC	
IQCG (chr3_197612220)	Outer-F: CTCCTGCTGCTCCTCAGACAT	RESS-qPCR
	Outer-R: TCCGCCTCCTGGGTTCA	
	WT-F: TAGCTGGGTGTGGTGGCTCGCA	
	Edit-R: CCTCCCAAAGTGCTGAGATTACATGC	
PDP2 (*chr16_66923766*)	Outer-F: GGGATTCCAAGACAGAAGACTCA	RESS-qPCR
	Outer-R: CACCGTGCACAGCCGACT	
	WT-F: TAGTCTCAGCTGCTTGGAAGGCTCAA	
	Edit-R: CTGGGCTCAAGAGATCCTCTCGTC	
CDK13 (*chr7_39990548*)	Outer-F: GCCGCCTCTGCTCTTCCT	RESS-qPCR
	Outer-R: CTCACATCCTCGTATTCCACCA	
	WT-F: AGGCGCGCAGGAGGGCGTCA	
	Edit-R: GCCCGCGACGCCGCTACC	
MFSD14A (*chr1_100489996*)	Outer-F: TGCCTGTAATCCCAGCACTTT	RESS-qPCR
	Outer-R: GGAGTCTCGCTCTGTCACCC	
	WT-F: GTGCGCGCCTGTAGTCCCAGTTA	
	Edit-R: TCTCCTGCCTCAGCCTCCTGTGC	
DNAJC24	Outer-F: GTCGCTGTAACCTCCGTCTCC	RESS-qPCR
	Outer-R: GGCTCACGCCTGCAATCC	

(*chr11_31452789*)	WT-F: ATGTTGGCCAGGCTGGTCTCGAA<br>Edit-R: AGGTGGATCACCTGAGGTCAGGTGC	
ENO4 (*chr10_118591949*)	Outer-F: TAGGCTGACCTCCTGAATGATG<br>Outer-R: CTCCCCAGTTCAAGCGATTC<br>WT-F: CACGGTGGCTCATGCCTGCAA<br>Edit-R: CTCAGCCTCCCAACATGCTGGTAC	RESS-qPCR
GLI4 (*chr8_144349912*)	Outer-F: GCCGTGACCTTGGCCTTG<br>Outer-R: CCTCTGCCCCTCTGTTCCC<br>WT-F: CAGGGCGCCTTTCCCAGTCA<br>Edit-R: ACGACAGCTGCCGTCACTGAGC	RESS-qPCR
CAPS (*chr19_5909721*)	Outer-F: GCAGTGGTGCGATTTTGGC<br>Outer-R: CCCTGAATGTCCTGGGCTAAG<br>WT-F: ACCATGTTGACCAGGCTGGTCACA<br>Edit-R: GGTGGATCACCTAAGGTCAGGAGGTC	RESS-qPCR
CLCC1 (*chr1_109475543*)	Outer-F: GGTGTCTGTCAGGATTCATTTGTT<br>Outer-R: TTTGGAAGGTTGAGGGAGGA<br>WT-F: GAATATATTAACATGTATAATTTTGAGAGAA<br>Edit-R: AGCCTGGGTGACAGAGCAAGAGCC	RESS-qPCR
CYCS (*chr7_25*	Outer-F: GTCAGGCGTTTGAGACCAGC<br>Outer-R: AGACAGTTTCACTCGTCACCCA	RESS-qPCR

159807)	WT-F: TGGGAGGCTGAGGCACAAGAAGCA	
	Edit-R: AACCTCCACCTCCCAAGTTCAGGC	
SYTL1 (*chr1_27678712*)	Outer-F: AGGGGGTTCCCAGGCTTT	RESS-qPCR
	Outer-R: TTTTTGAGATCGAGTCTCGCTCT	
	WT-F: GCGTGGTGGTGGGTGCCTGAAA	
	Edit-R: CGCCTCAGTCTCTCAAGTAGCTGGTAC	
PSPH (*chr7_56079465*)	Outer-F: TCATTTGTACCAACTTTCTATAGCAAG	RESS-qPCR
	Outer-R: GCCTGGGTGACAGAGCGA	
	WT-F: CTGTACGACAATGGATGTTATTCTTGCA	
	Edit-R: TGATTTTGTAGAGCTGCTGGGAGAGCC	
AMD1 (*chr6_111218862*)	Outer-F: ACCTCTACCTCCTGGGTTCAAG	RESS-qPCR
	Outer-R: AATTCTCCATGTTAAAACTGGGC	
	WT-F: GGGTTTCATCATGTTGGCCAGGTTA	
	Edit-R: ATCATCTGAGGTCAGGAGTTCCAGTCC	
SLC35A3 (*chr1_100440574*)	Outer-F: CCTAGCACAATGCCTGATATACTGT	RESS-qPCR
	Outer-R: AGAAGTCTGAGACCATCCTGGG	
	WT-F: AGTACAGAGGCGCAATCTCAGCACA	
	Edit-R: CCAGGGAGGTGGAAGTTGCTGC	
GNL3L (*chrX_54587219*)	Outer-F: TCTCACTTCCCTTCCGCTCC	RESS-qPCR
	Outer-R: TGCCTTAGTCTCCCAGGTTCAG	
	WT-F: TAGGTGCTGTGGCTCACGTCTGGAA	

	Edit-R: TCGGCCTCCCAAAGTGCTGGTAC	
GPR83 (*chr11_94150299*)	Outer-F: TATCTCAATGGGCTGGGTGC	RESS-qPCR
	Outer-R: GGCACAATCTCGGCTCACTG	
	WT-F: CTAAAAATACAAAAGATTAGCTGGCCA	
	Edit-R: AGCTAAGATTACAGGCACACACCACGAC	
DUSP21 (*chrX_44563168*)	Outer-F: ACGGAGTCTCGTTCTGTCGC	RESS-qPCR
	Outer-R: TTCATATTAAGAATCAAGGCTTTCAG	
	WT-F: CACCCACGTAATTTTTGTATTTTTACTA	
	Edit-R: AACATGGTGAAATCCCGTCACC	
SPAG9 (*chr17_49042216*)	Outer-F: TGGTGGCTCACGCCTGTAA	RESS-qPCR
	Outer-R: AGCAAGAAAATCAGTGAAATCCAG	
	WT-F: GGAGGCTGAGGTGGGAGGAGGA	
	Edit-R: CTCCACCTCCTGGGCCCATGC	
CBS (*chr21_44474331*)	Outer-F: GCTCACGCCTGTAATCCCA	RESS-qPCR
	Outer-R: AGCGGCGAAGGCTGTGT	
	WT-F: GAGTCTGAGGCACGAGAATCACTTCAA	
	Edit-R: TCACTGCAACCTCCACCTCCTGAGC	
ARMC5 (*chr16_31465937*)	Outer-F: GACTACAGGCATACACCACCACA	RESS-qPCR
	Outer-R: ACAACAAACCAAGCCCCAGA	
	WT-F: GTTCCTCTGGTCTCAGCCTCCCGAA	
	Edit-R: TGGCTCACATCTGTAATCCCAGCGTC	

Supplementary Table 3. Recombinant proteins used for this study.

Protein Name	Source	Application
EZH2 protein with N-His/GST tag	BPS Bioscience, 50279	AlphaLISA, GST Pulldown
EZH2 protein with N-GST tag	Creative Biomart, EZH2-285H	AlphaLISA
ADAR1 protein with C-Myc/Flag tag	ORIGENE, TP319761	AlphaLISA, GST Pulldown
ADAR2 protein with C-Myc/Flag tag	ORIGENE, TP312324	AlphaLISA, GST Pulldown
ILF2 protein with N-His tag	Creative Biomart, ILF2-29033TH	AlphaLISA
