# Supplementary material for: A dual role of EZH2 in regulating A-to-I RNA editing and mRNA stability through ADAR
Source: Nat Commun. 2026 Mar 26;17:4421. doi: 10.1038/s41467-026-71207-3 (PMC13184275; doi:10.1038/s41467-026-71207-3)
Supplement: Supplementary file 2 — Description of additional supplementary files [file 41467_2026_71207_MOESM2_ESM.pdf]

Description of Additional Supplementary Files

File name: Supplementary Data 1

Description: List of editing sites with altered editing ratios upon EZH2, ADAR1 or ADAR2 knockdown in C4-2 cells

File name: Supplementary Data 2

Description: Summary of EZH2-affected editing sites verification results

File name: Supplementary Data 3

Description: Summary of EZH2, ILF2 and ADAR1-binding RNA targets based on eCLIP-seq results

File name: Supplementary Data 4

Description: Summary of genes with altered RNA half-lives upon EZH2 or ADAR1 deficiency
