# Supplementary material for: A dual role of EZH2 in regulating A-to-I RNA editing and mRNA stability through ADAR
Source: Nat Commun. 2026 Mar 26;17:4421. doi: 10.1038/s41467-026-71207-3 (PMC13184275; doi:10.1038/s41467-026-71207-3)
Supplement: Supplementary file 7 — Reporting Summary [file 41467_2026_71207_MOESM7_ESM.pdf]

nature portfolio

Corresponding author(s): Qi Cao and Kaifu Chen

Last updated by author(s): Feb 16, 2026

Reporting Summary

Nature Portfolio wishes to improve the reproducibility of the work that we publish. This form provides structure for consistency and transparency in reporting. For further information on Nature Portfolio policies, see our Editorial Policies and the Editorial Policy Checklist.

Statistics

For all statistical analyses, confirm that the following items are present in the figure legend, table legend, main text, or Methods section.

n/a	Confirmed	
☐	☒	The exact sample size (*n*) for each experimental group/condition, given as a discrete number and unit of measurement
☐	☒	A statement on whether measurements were taken from distinct samples or whether the same sample was measured repeatedly
☐	☒	The statistical test(s) used AND whether they are one- or two-sided<br>*Only common tests should be described solely by name; describe more complex techniques in the Methods section.*
☐	☒	A description of all covariates tested
☐	☒	A description of any assumptions or corrections, such as tests of normality and adjustment for multiple comparisons
☐	☒	A full description of the statistical parameters including central tendency (e.g. means) or other basic estimates (e.g. regression coefficient) AND variation (e.g. standard deviation) or associated estimates of uncertainty (e.g. confidence intervals)
☐	☒	For null hypothesis testing, the test statistic (e.g. *F*, *t*, *r*) with confidence intervals, effect sizes, degrees of freedom and *P* value noted<br>*Give P values as exact values whenever suitable.*
☒	☐	For Bayesian analysis, information on the choice of priors and Markov chain Monte Carlo settings
☒	☐	For hierarchical and complex designs, identification of the appropriate level for tests and full reporting of outcomes
☐	☒	Estimates of effect sizes (e.g. Cohen's *d*, Pearson's *r*), indicating how they were calculated

Our web collection on statistics for biologists contains articles on many of the points above.

Software and code

Policy information about availability of computer code

Data collection	Confocal Images were acquired with the aid of Nikon software (NIS-Elements, v 4.50). Immunohistochemistry (IHC) and Proximity ligation assay (PLA) slides were read by Aperio ImageScope software (ImageScope v. 12.4). The transcriptome sequencing and matched whole exome sequencing data of Stand Up To Cancer (SU2C) metastatic prostate cancer (PCa) samples were from the dbGAP dataset (phs000915.v2p2).The datasets of EHZ2 knockdown samples of LnCAP, LnCAP-Abl, HL-60, MCF7, and SUM149 were downloaded from GEO database. BAM files from 552 prostate adenocarcinoma (PRAD) patients were downloaded through gdc-client v1.6.1 from TCGA database.
Data analysis	The domain structure of protein was illustrated using Domain Graph (DOG) 2.0 software.<br>Statistics were analyzed using Prism (v. 6.0).<br>Analysis of images was performed using Image J v. 2.0.<br>For RNA-seq, the raw reads with fastq format were aligned to the human reference genome hg19 using STAR aligner (v2.7.9a). The count number of mapped reads was calculated by htseq-count (v0.11.2). After mapping, the software of REDItoolDnaRna.py from REDItools (v1.3) was applied to detect the RNA editing events.<br>For eCLIP-seq analysis, both STAR (v2.7.9a) with default parameters and UCSC known genes were firstly employed to map the genome hg19, followed by peak calling using CLIP-seq peak caller Piranha with default parameters and bin size=20. To define the RBP binding peaks in each sample, a P-value cutoff of 0.01 was set up. The homer (v4.8.3) annotatePeaks.pl script was then applied to annotate the peaks from the eCLIP-seq data.<br>A down sampling step was performed using the samtools view function to match the sample with the smallest read count. The software bamCoverage from deeptools (v3.5.5) was used to generate a coverage track bigWig as output, the bigwigCompare was used to generate the input substracted bigwig signal. A similar analysis was conducted on the ADAR RNA immunoprecipitation sequencing (RIP-seq) dataset. The software htseq-count (v0.11.2) was used to quantify raw reads mapped to each gene, and the log2 fold change of RIP/input based on RPM

(reads per million) was calculated to compare control and EZH2 knockdown samples. The authors does generate custom algorithms or software for this manuscript.

For manuscripts utilizing custom algorithms or software that are central to the research but not yet described in published literature, software must be made available to editors and reviewers. We strongly encourage code deposition in a community repository (e.g. GitHub). See the Nature Portfolio guidelines for submitting code & software for further information.

Data

Policy information about availability of data

All manuscripts must include a data availability statement. This statement should provide the following information, where applicable:

- Accession codes, unique identifiers, or web links for publicly available datasets
- A description of any restrictions on data availability
- For clinical datasets or third party data, please ensure that the statement adheres to our policy

The next-generation sequencing data that support the findings of this study have been deposited in the Gene Expression Omnibus (GEO) under accession code of GSE225951.

Research involving human participants, their data, or biological material

Policy information about studies with human participants or human data. See also policy information about sex, gender (identity/presentation), and sexual orientation and race, ethnicity and racism.

Reporting on sex and gender	All the human specimen were obtained from male patients since we focus on prostate cancer.
Reporting on race, ethnicity, or other socially relevant groupings	We had no access to ethnicity or any lifestyle data except the patient's hospital record associated with the clinical follow-up data of potential treatment after surgery and mortality. The patients used in this cohort had no records of additional genetic background.
Population characteristics	All the human specimen were obtained from Vancouver Prostate Centre Tissue bank. This study applied a Tissue Micro-array constructed from 80 radical prostectomys (RP) and transurethral resection of the prostate(TURPs).
Recruitment	There was no patient recruitment for this study specifically. We have used master clinical data of VPC tissue bank to select the specimens. TMA constructed using 22 RPs with no prior exposure to treatment as controls, 13 RPs with neoadjuvant therapy prior to surgery and 45 TURP specimens to study the progression and trans-differentiation of the tumors. We tried to minimize the bias by comparing 4 groups based on their immunoreactivity against neuroendocrine markers.
Ethics oversight	The University of British Columbia, Office of Research Ethics, Clinical Research Ethics Board , UBC CREB NUMBER: H09-01628

Note that full information on the approval of the study protocol must also be provided in the manuscript.

Field-specific reporting

Please select the one below that is the best fit for your research. If you are not sure, read the appropriate sections before making your selection.

☒ Life sciences ☐ Behavioural & social sciences ☐ Ecological, evolutionary & environmental sciences

For a reference copy of the document with all sections, see nature.com/documents/nr-reporting-summary-flat.pdf

Life sciences study design

All studies must disclose on these points even when the disclosure is negative.

Sample size	Sample sizes were chosen to reliably measure experimental parameters while keeping with standards in the relevant fields, and remaining in compliance with ethical guidelines to minimize the number of animals used.
Data exclusions	No data were excluded from analysis.
Replication	All data was reliably reproduced. The number of repeats is indicated in each figure legend.
Randomization	Randomization was used with respect to time point of data collection.
Blinding	Experiments did not involve blinding, but randomization was used with respect to time point of data collection. Animal subjects were randomly allocated to each group.

Reporting for specific materials, systems and methods

We require information from authors about some types of materials, experimental systems and methods used in many studies. Here, indicate whether each material, system or method listed is relevant to your study. If you are not sure if a list item applies to your research, read the appropriate section before selecting a response.

Materials & experimental systems

n/a	Involved in the study
☐	☒ Antibodies
☐	☒ Eukaryotic cell lines
☒	☐ Palaeontology and archaeology
☐	☒ Animals and other organisms
☒	☐ Clinical data
☒	☐ Dual use research of concern
☒	☐ Plants

Methods

n/a	Involved in the study
☒	☐ ChIP-seq
☒	☐ Flow cytometry
☒	☐ MRI-based neuroimaging

Antibodies

Antibodies used	Please see Supplementary Table 1 "Antibodies used in this study" for details.
Validation	All antibodies are commercially available and validated by the manufacturers for use in human.

Eukaryotic cell lines

Policy information about cell lines and Sex and Gender in Research

Cell line source(s)	Human PCa cell line C4-2 was a generous gift from Dr. Leland Chung (Cedars-Sinai), while PC-3 was purchased from ATCC. Human Prostate Epithelial Cells (PrEC) were purchased from Lonza. HEK293T cells were purchased from ATCC.
Authentication	All the cell lines used in this study were authenticated by STR profiling.
Mycoplasma contamination	All the cell lines used in this study were tested negative for mycoplasma contamination.
Commonly misidentified lines (See ICLAC register)	No commonly misidentified cell lines were used in the study.

Animals and other research organisms

Policy information about studies involving animals; ARRIVE guidelines recommended for reporting animal research, and Sex and Gender in Research

Laboratory animals	Five-week-old male SCID mice were used in this study.
Wild animals	No wild animals were used in the study.
Reporting on sex	Only male mice were used since our study focuses on prostate cancer, a male cancer type.
Field-collected samples	No field collected samples were used in the study.
Ethics oversight	Animal care and use conditions were followed in accordance with institutional and National Institutes of Health protocols and guidelines, and all studies were approved by Northwestern University Animal Care and Use Committee.

Note that full information on the approval of the study protocol must also be provided in the manuscript.

Plants

Seed stocks	na
Novel plant genotypes	na
Authentication	na
