# Supplementary material for: A dual role of EZH2 in regulating A-to-I RNA editing and mRNA stability through ADAR
Source: Nat Commun. 2026 Mar 26;17:4421. doi: 10.1038/s41467-026-71207-3 (PMC13184275; doi:10.1038/s41467-026-71207-3)

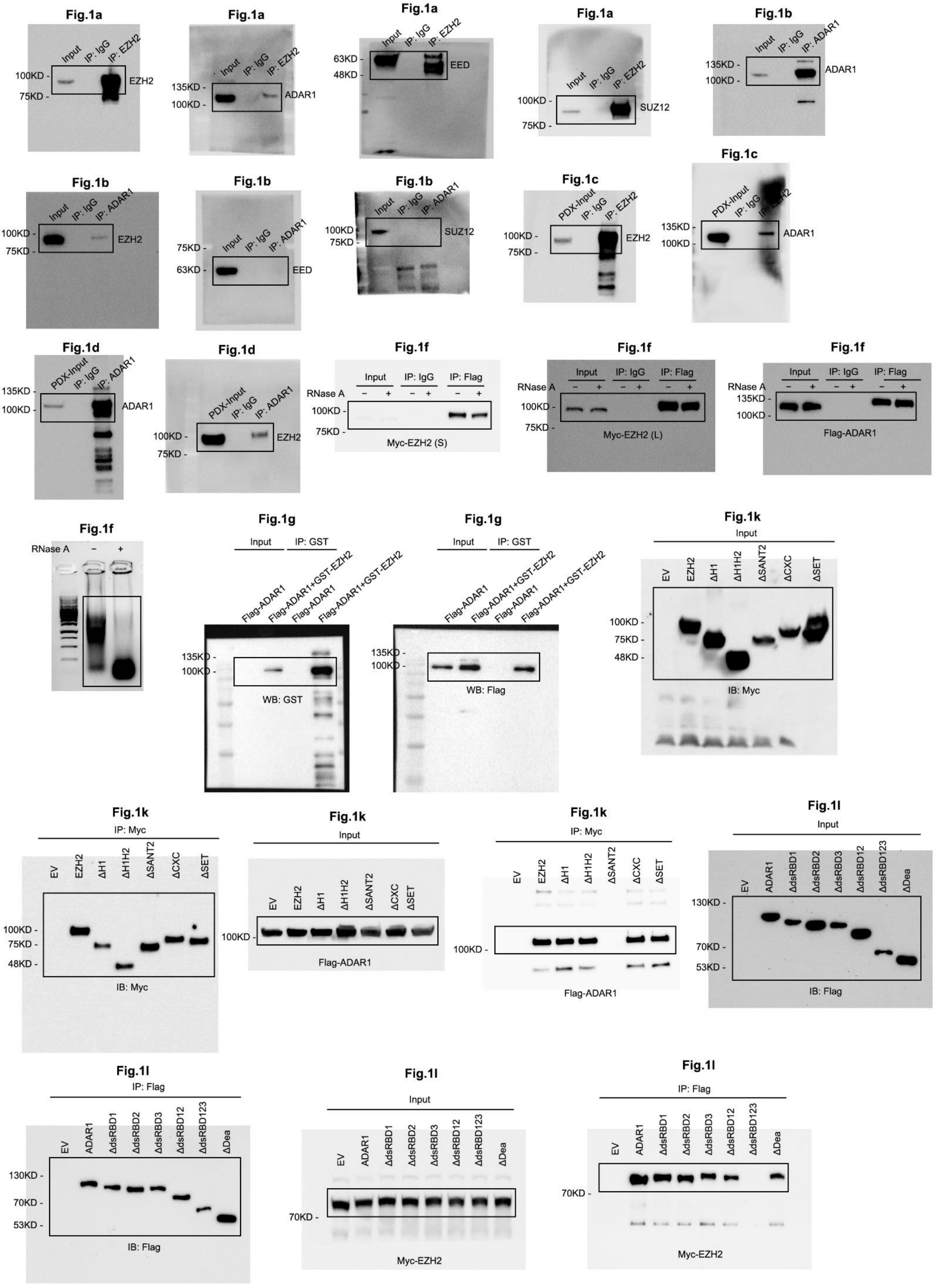
Fig.1a
Input
IP: IgG
IP: EZH2
100KD -
75KD -
EZH2
Fig.1a
Input
IP: IgG
IP: EZH2
135KD -
100KD -
ADAR1
Fig.1a
Input
IP: IgG
IP: EZH2
63KD -
48KD -
EED
Fig.1a
Input
IP: IgG
IP: EZH2
100KD -
75KD -
SUZ12
Fig.1b
Input
IP: IgG
IP: ADAR1
135KD -
100KD -
ADAR1
Fig.1b
Input
IP: IgG
IP: ADAR1
100KD -
75KD -
EZH2
Fig.1b
Input
IP: IgG
IP: ADAR1
75KD -
63KD -
EED
Fig.1b
Input
IP: IgG
IP: ADAR1
100KD -
75KD -
SUZ12
Fig.1c
PDX-Input
IP: IgG
IP: EZH2
100KD -
75KD -
EZH2
Fig.1c
PDX-Input
IP: IgG
IP: EZH2
135KD -
100KD -
ADAR1
Fig.1d
PDX-Input
IP: IgG
IP: ADAR1
135KD -
100KD -
ADAR1
Fig.1d
PDX-Input
IP: IgG
IP: ADAR1
100KD -
75KD -
EZH2
Fig.1f
Input
IP: IgG
IP: Flag
RNase A
100KD -
75KD -
Myc-EZH2 (S)
Fig.1f
Input
IP: IgG
IP: Flag
RNase A
100KD -
75KD -
Myc-EZH2 (L)
Fig.1f
Input
IP: IgG
IP: Flag
RNase A
135KD -
100KD -
Flag-ADAR1
Fig.1f
RNase A
Fig.1g
Input
IP: GST
Flag-ADAR1
Flag-ADAR1+GST-EZH2
Flag-ADAR1
Flag-ADAR1+GST-EZH2
135KD -
100KD -
WB: GST
Fig.1g
Input
IP: GST
Flag-ADAR1
Flag-ADAR1+GST-EZH2
Flag-ADAR1
Flag-ADAR1+GST-EZH2
135KD -
100KD -
WB: Flag
Fig.1k
Input
EV
EZH2
ΔH1
ΔH1H2
ΔSANT2
ΔCXC
ΔSET
100KD -
75KD -
48KD -
IB: Myc
Fig.1k
IP: Myc
EV
EZH2
ΔH1
ΔH1H2
ΔSANT2
ΔCXC
ΔSET
100KD -
75KD -
48KD -
IB: Myc
Fig.1k
Input
EV
EZH2
ΔH1
ΔH1H2
ΔSANT2
ΔCXC
ΔSET
100KD -
Flag-ADAR1
Fig.1k
IP: Myc
EV
EZH2
ΔH1
ΔH1H2
ΔSANT2
ΔCXC
ΔSET
100KD -
Flag-ADAR1
Fig.1l
Input
EV
ADAR1
ΔdsRBD1
ΔdsRBD2
ΔdsRBD3
ΔdsRBD12
ΔdsRBD123
ΔDea
130KD -
70KD -
53KD -
IB: Flag
Fig.1l
IP: Flag
EV
ADAR1
ΔdsRBD1
ΔdsRBD2
ΔdsRBD3
ΔdsRBD12
ΔdsRBD123
ΔDea
130KD -
70KD -
53KD -
IB: Flag
Fig.1l
Input
EV
ADAR1
ΔdsRBD1
ΔdsRBD2
ΔdsRBD3
ΔdsRBD12
ΔdsRBD123
ΔDea
70KD -
Myc-EZH2
Fig.1l
IP: Flag
EV
ADAR1
ΔdsRBD1
ΔdsRBD2
ΔdsRBD3
ΔdsRBD12
ΔdsRBD123
ΔDea
70KD -
Myc-EZH2

Fig.S1a Fig.S1a Fig.S1a Fig.S1a

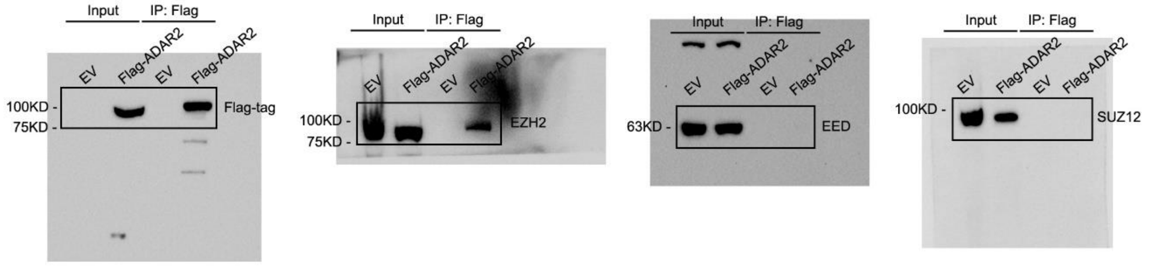

Fig.S1b Fig.S1b Fig.S1b Fig.S1b

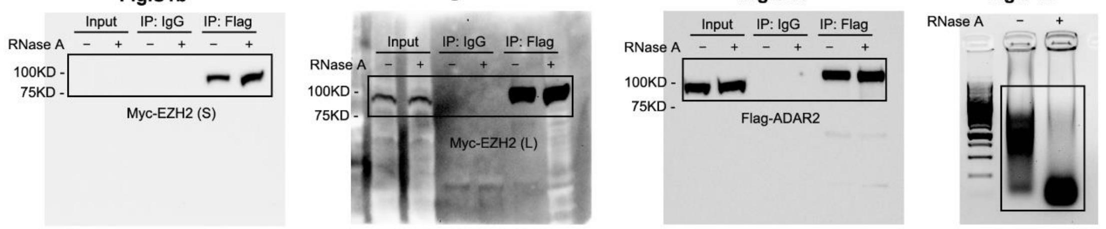

Fig.S1c Fig.S1c Fig.S1h

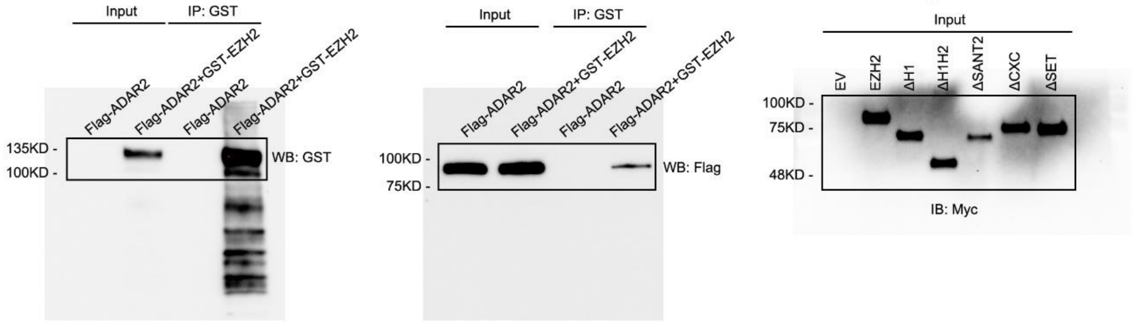

Fig.S1h Fig.S1h Fig.S1h

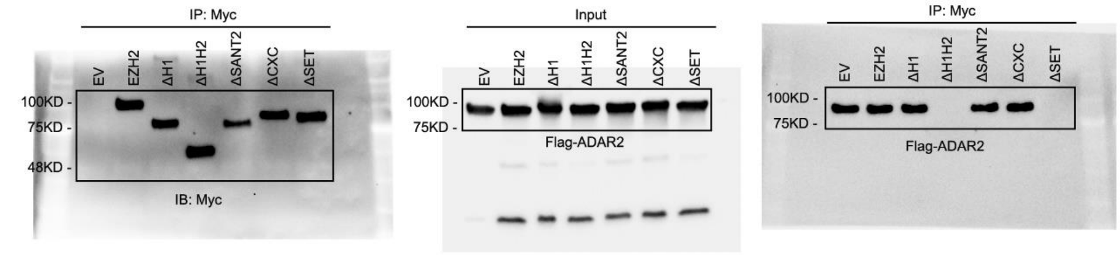

Fig.S1i Fig.S1i Fig.S1i Fig.S1i

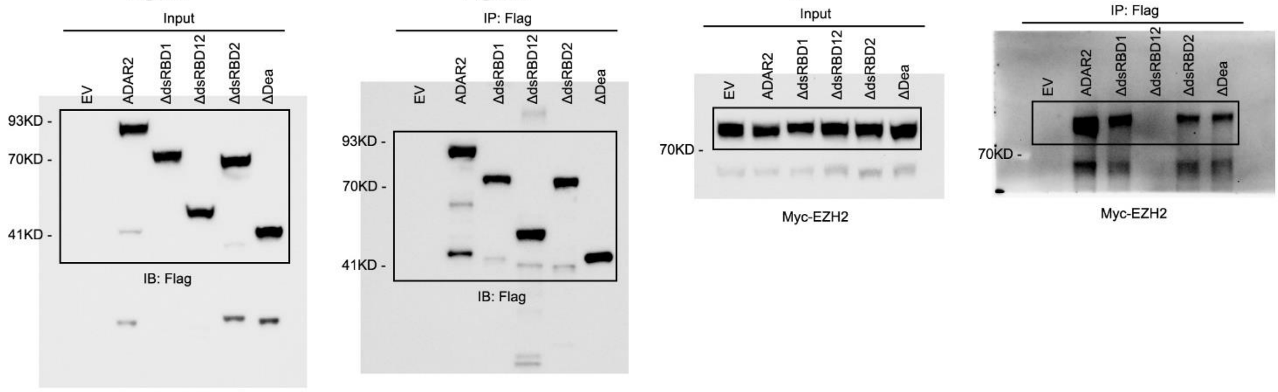

Fig.S2j

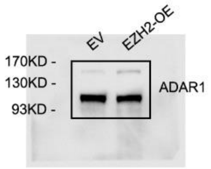

Fig.S2j

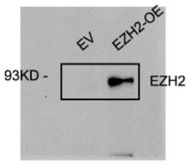

Fig.S2j

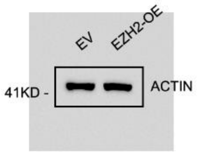

Fig.S2j

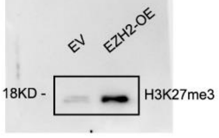

Fig.S2j

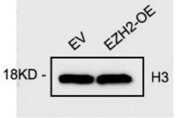

Fig.3g

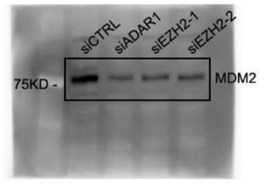

Fig.3g

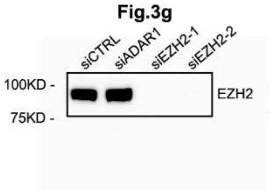

Fig.3g

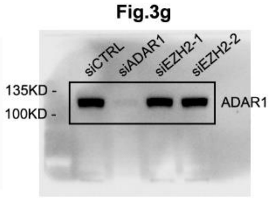

Fig.3g

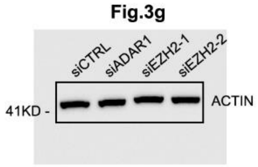

Fig.3i

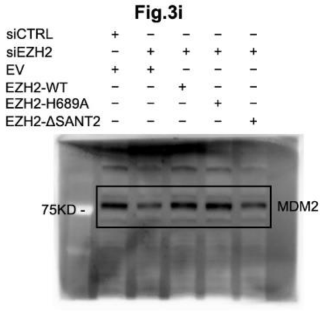

Fig.3i

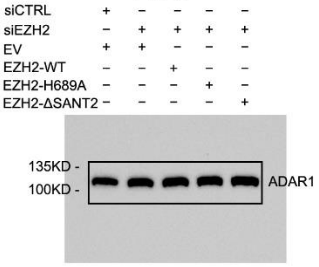

Fig.3i

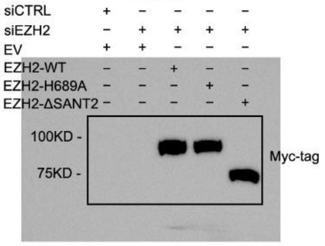

Fig.3i

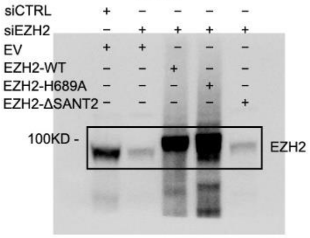

Fig.3i

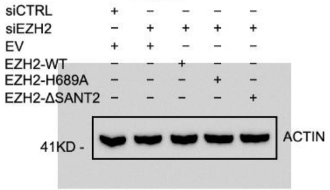

Fig.3i

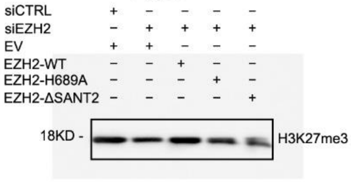

Fig.3i

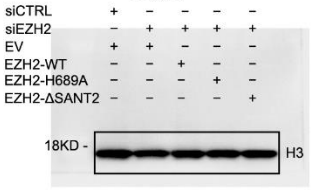

Fig.S3e

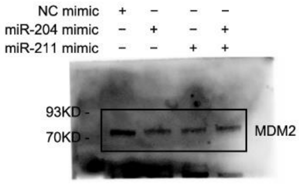

Fig.S3e

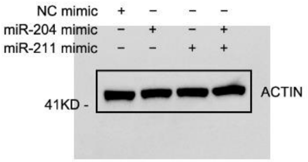

Fig.S3g

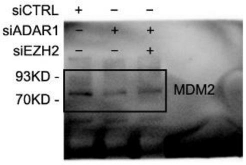

Fig.S3g

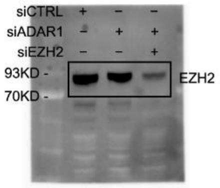

Fig.S3g

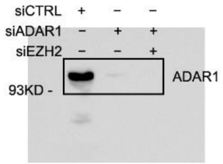

Fig.S3g

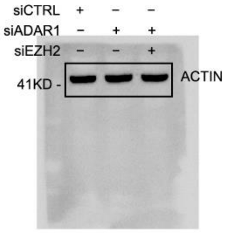

Fig.4b

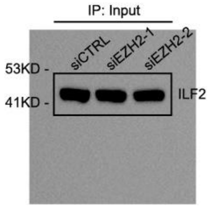

Fig.4b

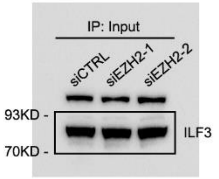

Fig.4b

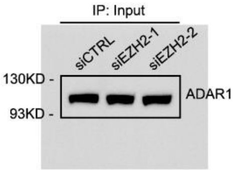

Fig.4b

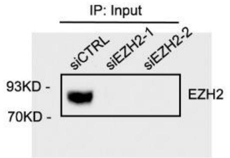

Fig.4b

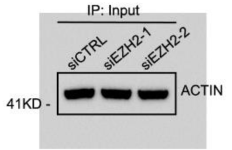

Fig.4b

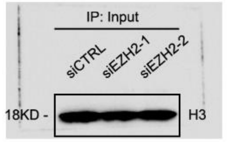

Fig.4b

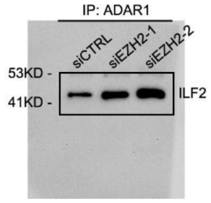

Fig.4b

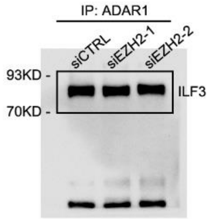

Fig.4b

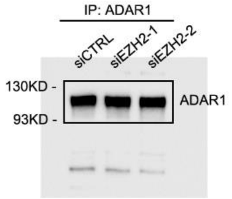

Fig.4c

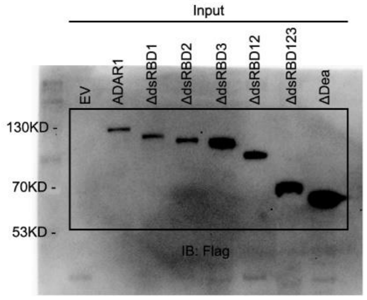

Fig.4c

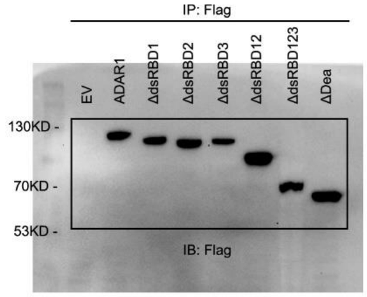

Fig.4c

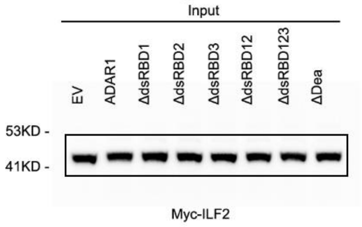

Fig.4c

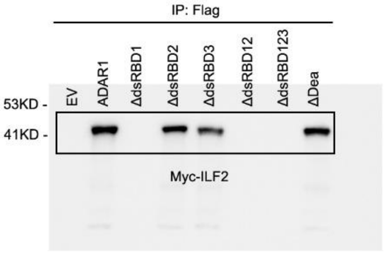

Fig.4e

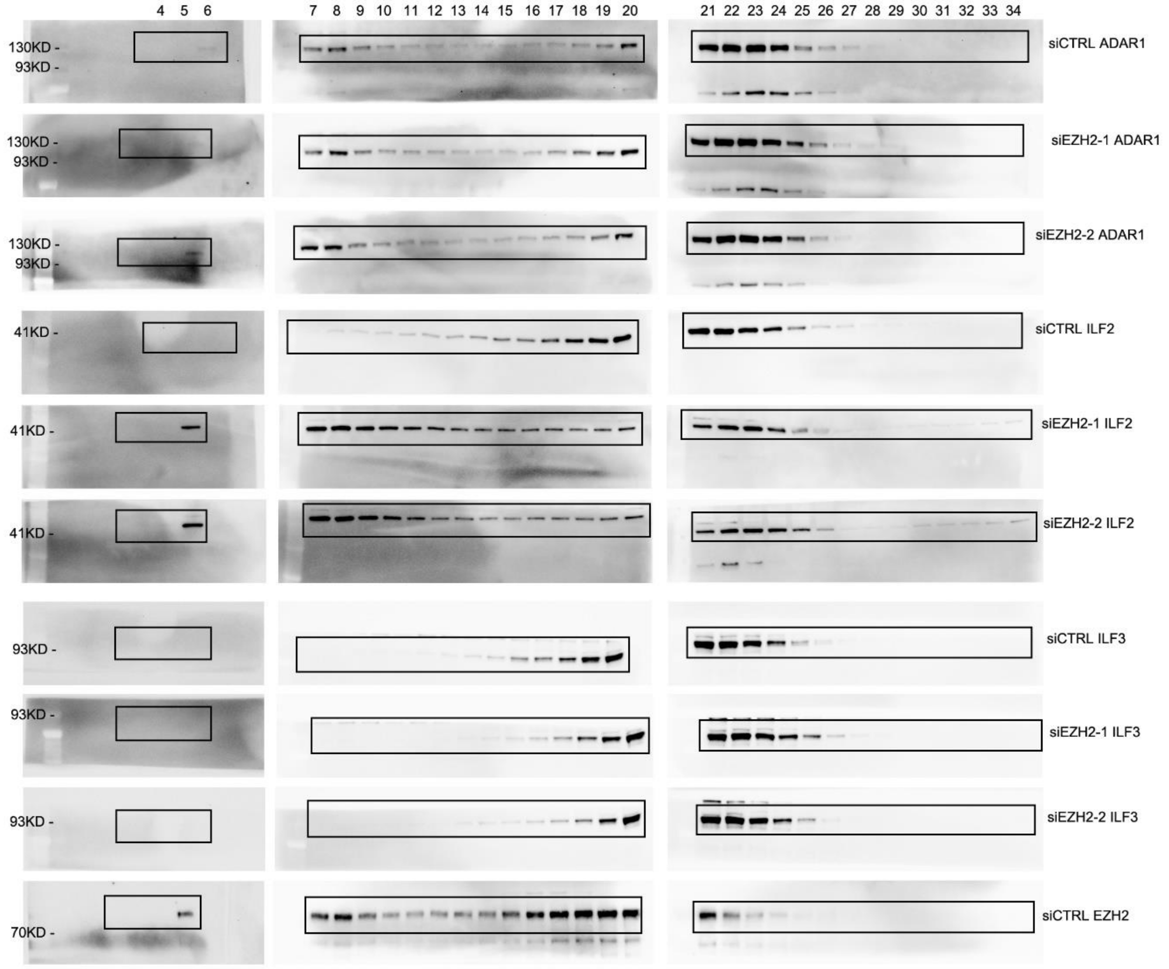

Fig.S4a

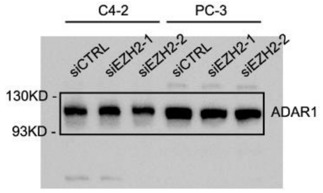

Fig.S4a

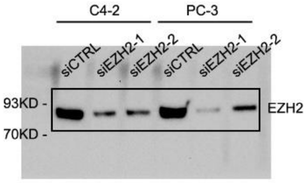

Fig.S4a

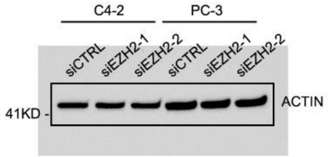

Fig.S4a

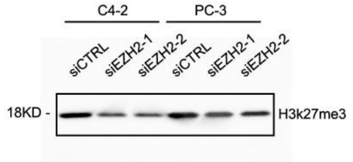

Fig.S4a

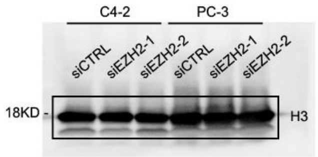

Fig.S4b

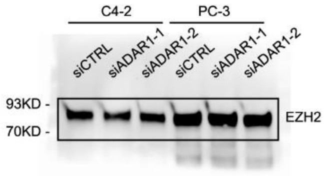

Fig.S4b

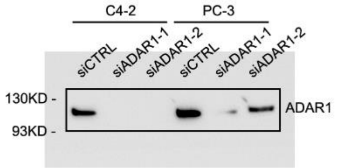

Fig.S4b

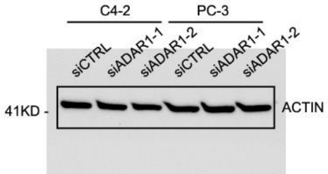

Fig.S4b

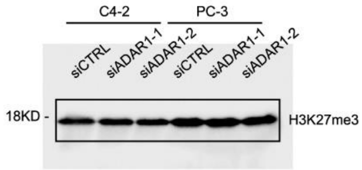

Fig.S4b

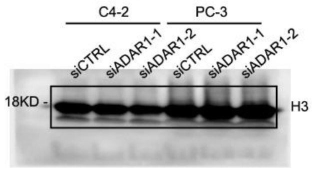

Fig.S4c

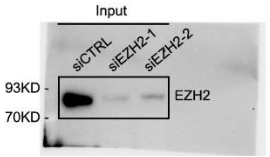

Fig.S4c

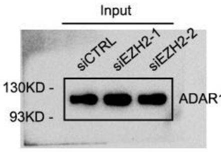

Fig.S4c

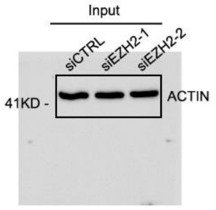

Fig.S4c

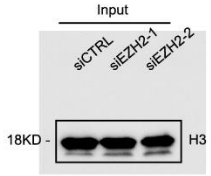

Fig.S4c

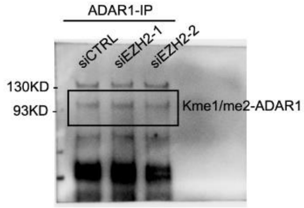

Fig.S4c

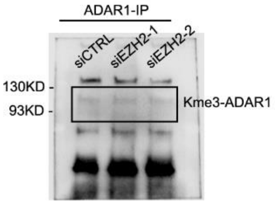

Fig.S4c

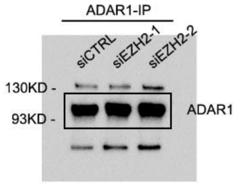

Fig.S4d

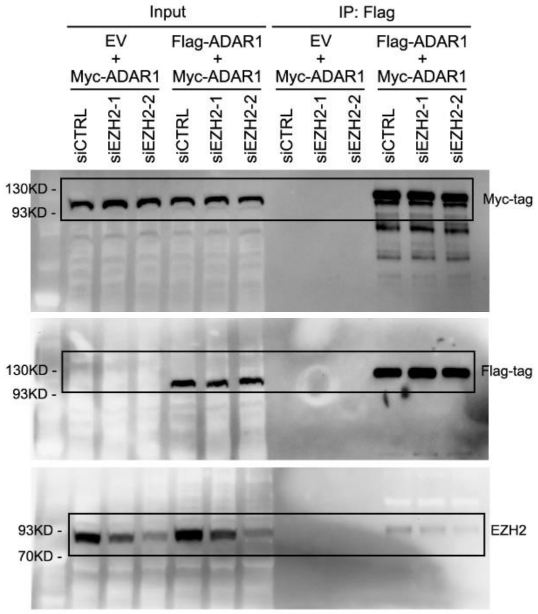

Fig.S4f

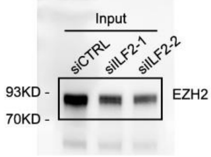

Fig.S4f

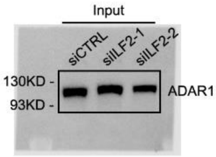

Fig.S4f

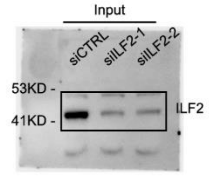

Fig.S4f

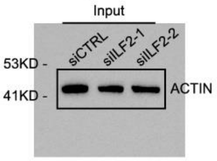

Fig.S4f

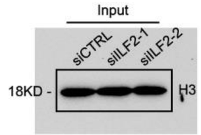

Fig.S4f

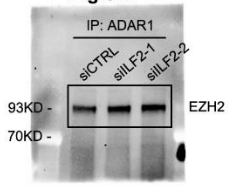

Fig.S4f

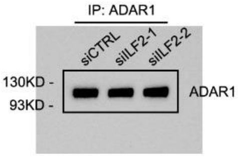

Fig.5a

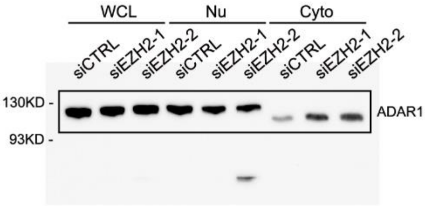

Fig.5a

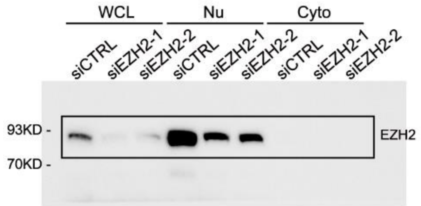

Fig.5a

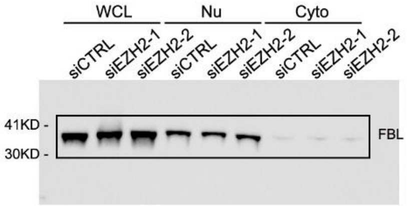

Fig.5a

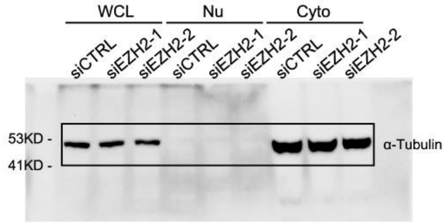

Fig.5c

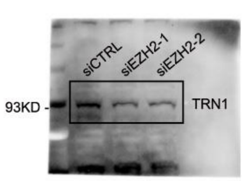

Fig.5c

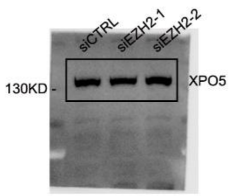

Fig.5c

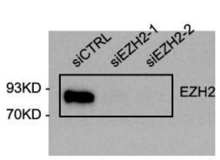

Fig.5c

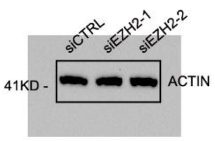

Fig.5c

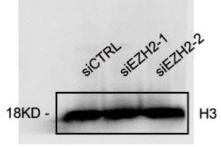

Fig.5d

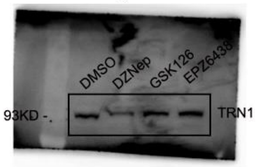

Fig.5d

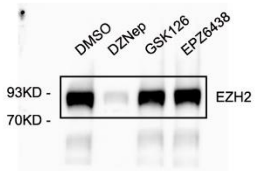

Fig.5d

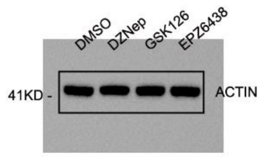

Fig.5d

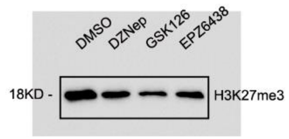

Fig.5d

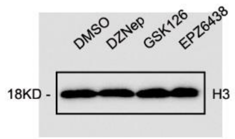

Fig.5j

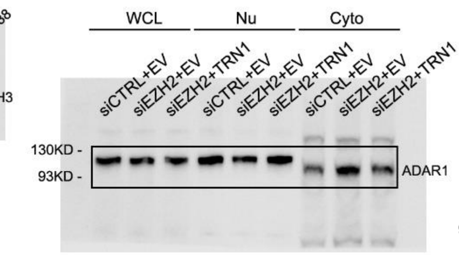

Fig.5j

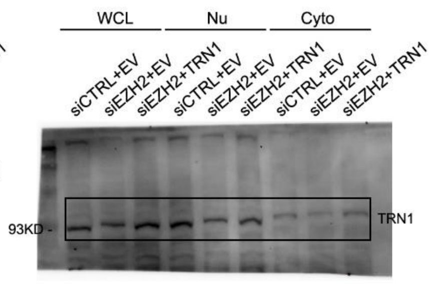

Fig.5j

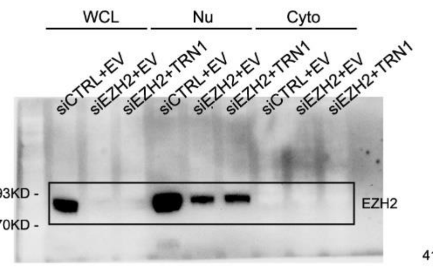

Fig.5j

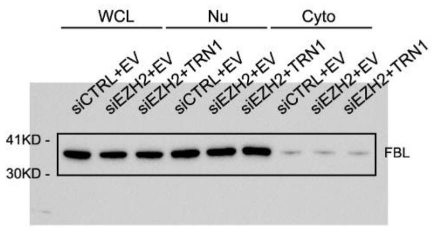

Fig.5j

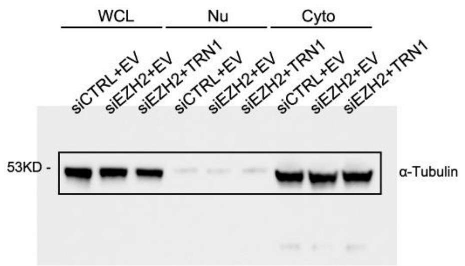

Fig.S5a

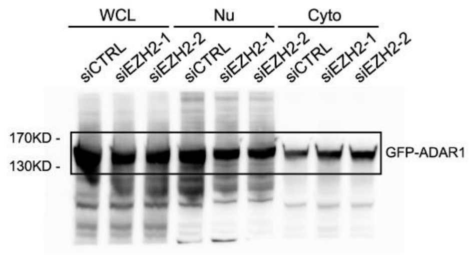

Fig.S5a

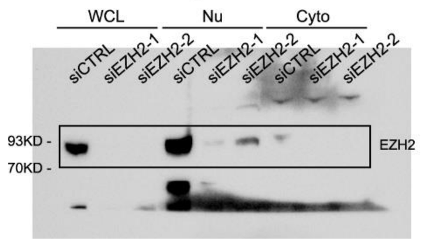

Fig.S5a

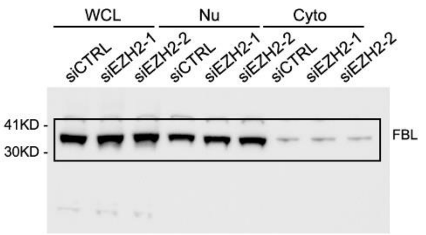

Fig.S5a

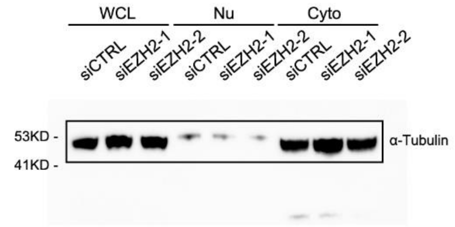

Fig.S5c

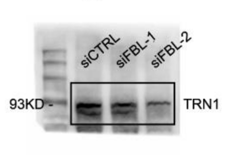

Fig.S5c

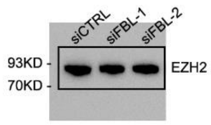

Fig.S5c

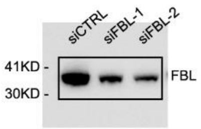

Fig.S5c

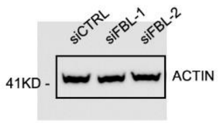

Fig.6g

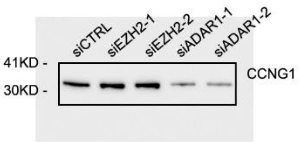

Fig.6g

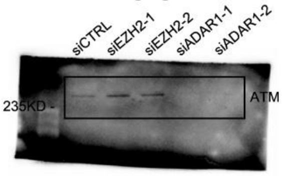

Fig.6g

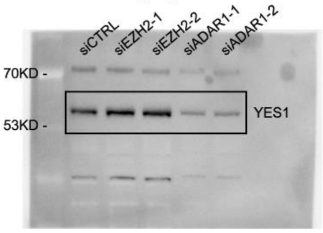

Fig.6g

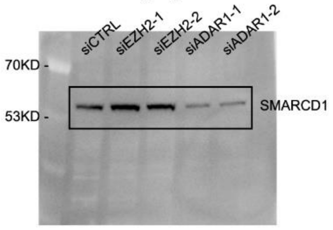

Fig.6g

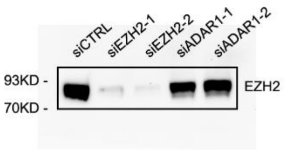

Fig.6g

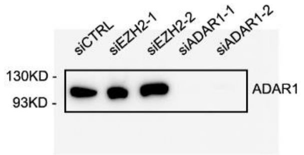

Fig.6g

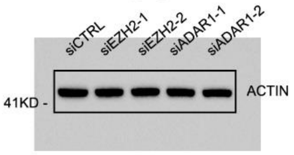

Fig.6h

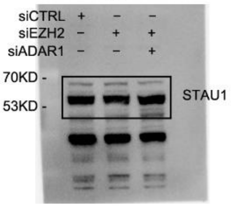

Fig.6h

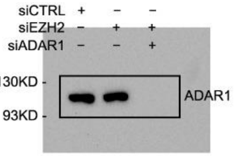

Fig.6h

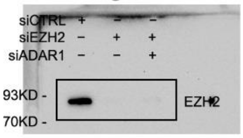

Fig.6h

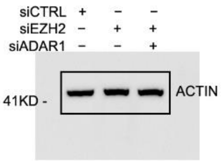

Fig.7a

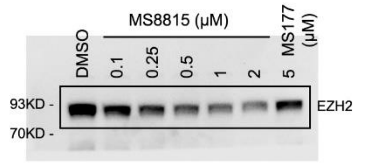

Fig.7a

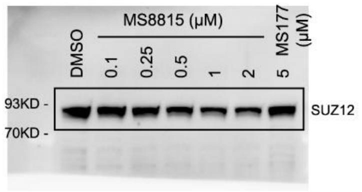

Fig.7a

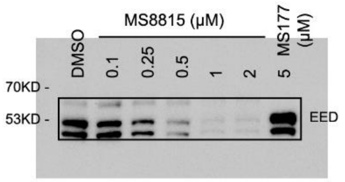

Fig.7a

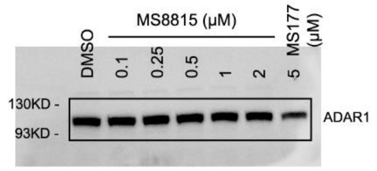

Fig.7a

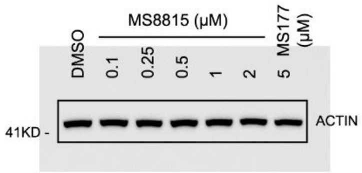

Fig.7a

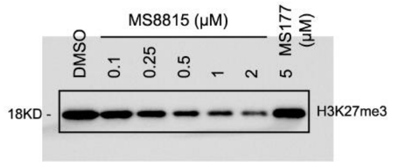

Fig.7a

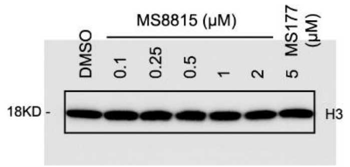

Fig.7b

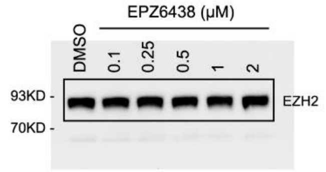

Fig.7b

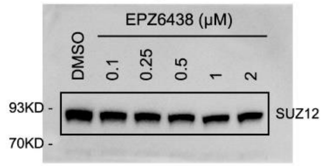

Fig.7b

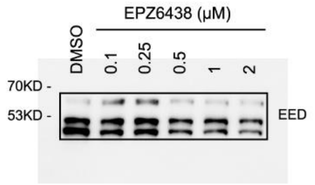

Fig.7b

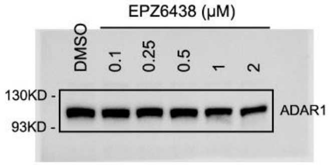

Fig.7b

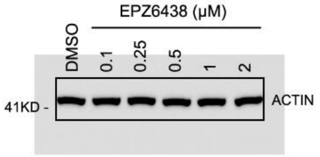

Fig.7b

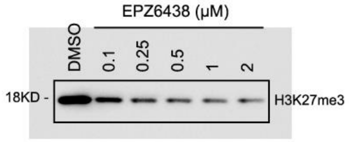

Fig.7b

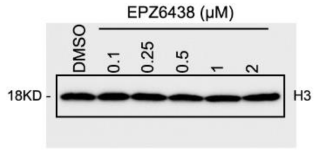

Fig.S7d

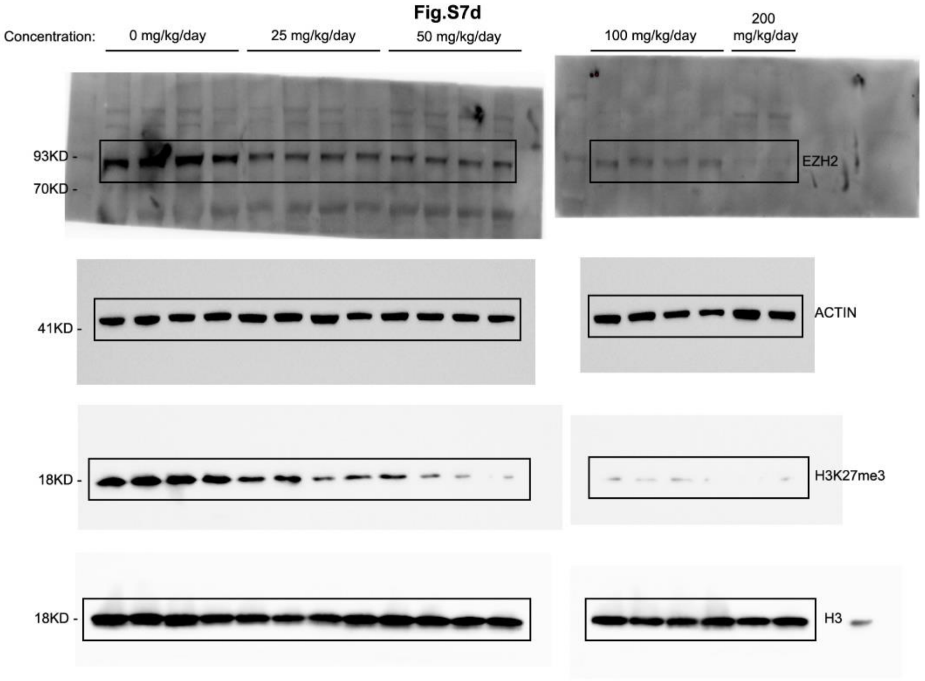

Fig.S7e

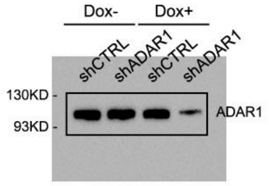

Fig.S7e

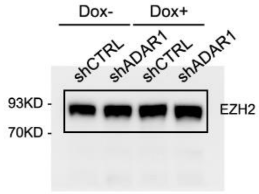

Fig.S7e

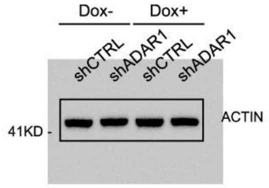

Fig.S7f

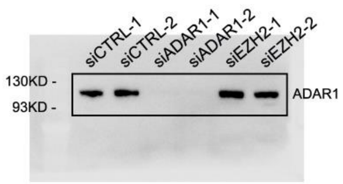

Fig.S7f

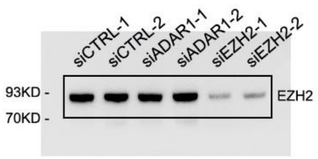

Fig.S7f

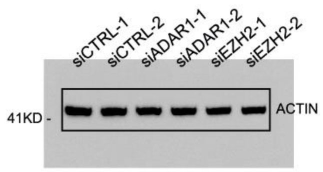

Supplement: Supplementary file 9 — Source Data [file 41467_2026_71207_MOESM9_ESM.zip › Raw image source data.pdf]
